# Paraoxonase-1 polymorphisms and cerebral ischemic stroke: a pilot study in mexican patients

**DOI:** 10.25100/cm.v49i2.2217

**Published:** 2018-09-30

**Authors:** María Fernanda Martínez-Salazar, María de la Luz Soriano-Martínez, Alina Juantorena-Ugas, Damianys Almenares-López, Petra Yescas, Marie-Catherine Boll, Antonio Monroy-Noyola

**Affiliations:** 1 Laboratorio de Bioquímica, Facultad de Ciencias del Deporte, Universidad Autónoma del Estado de Morelos. Cuernavaca, México; 2 Laboratorio de Neuroprotección, Facultad de Farmacia, Universidad Autónoma del Estado de Morelos. Cuernavaca, México; 3 División Ciencias Agropecuarias e Ingenierías, Universidad Popular de la Chontalpa, Cárdenas, Tabasco, México; 4 Departamento de Neurogenética, Instituto Nacional de Neurología y Neurocirugía, M.V.S. Ciudad de México. Mexico; 5 Investigación Clínica, Instituto Nacional de Neurología y Neurocirugía, M.V.S. Ciudad de México México.

**Keywords:** Atherosclerosis, stroke, aryldialkylphosphatase, single nucleotide polymorphism, arylesterase, Carboxylic Ester Hydrolases, Ateroesclerosis, Cerebral. Infarto, Arildialquifosfatasa, Polimorfismos de nucleotidos únicos, arilesterasa, hidrolasas de éster carboxílico

## Abstract

**Background:**

The serum paraoxonase-1 (PON1) associated to HDL presents two common polymorphisms in the positions 192 and 55. These polymorphisms are considered determinant of the capacity of HDL to protect LDL from their oxidative modification. In this context, the PON1 genotype has been associated with cardiovascular diseases, including stroke.

**Objective:**

To determine the allelic and genotypic frequencies of PON1 L55M and Q192R as well as the enzymatic activities of PON1 in subjects with and without atherothrombotic stroke.

**Methods:**

There were included 28 people with atherothrombotic stroke and 29 without stroke. The genotyping was carried out by PCR-RFLP and the phenotyping by measurement of the activities of paraoxonase and arylesterase in serum.

**Results:**

For the polymorphism Q192R, the allelic frequencies (Q/R) were 0.46/0.54 and 0.48/0.52 (*p*= 0.843) for the control group and the group with stroke, respectively. While for the polymorphism L55M, the allelic frequencies (L/M) were 0.81/0.19 for the control group, and 0.78/0.22 for the group with stroke (*p*= 0.610). The activity levels of paraoxonase were not significantly different between the control and stroke groups (450 vs. 348 UI/mL, *p*= 0.093) While the activity levels of arylesterase were significantly different between the studied groups (90 vs. 70 UI/mL, *p*= 0.001); however, upon adjustment by multiple linear regression, it was not longer significant.

**Conclusion:**

The polymorphisms Q192R and L55M, and the paraoxonase activity of PON1 are not risk factors for atherothrombotic stroke according to the results of this study.

## Resumen

**Introducción:**

La paraoxonasa-1 (PON1) sérica asociada a las HDL presenta dos polimorfismos comunes en las posiciones 192 y 55. Estos polimorfismos se consideran determinantes para la capacidad de las HDL de proteger a las LDL de su modificación oxidativa. En este contexto, el genotipo de PON1 se ha asociado con enfermedades cerebrovasculares, que incluyen el infarto cerebral.

**Objetivo:**

Determinar las frecuencias alélicas y genotípicas de PON1-L55M y PON1- Q192R, así como las actividades enzimáticas de PON1 en sujetos con y sin infarto cerebral aterotrombótico.

**Métodos:**

Se incluyeron 28 personas con infarto cerebral aterotrombótico y 29 sin infarto. Las genotipificaciones se realizaron mediante PCR-RFLP y las fenotipificaciones mediante la medición de las actividades paraoxonasa y arilesterasa en suero.

**Resultados:**

Para el polimorfismo Q192R, las frecuencias alélicas (Q/R) fueron 0.46/0.54 y 0.48/0.52 (*p*= 0.843) para el grupo control y el grupo con infarto, respectivamente. Mientras que para el polimorfismo L55M, las frecuencias alélicas (L/M) fueron 0.81/0.19 para el grupo control y 0.78/0.22 para el grupo con infarto (*p*= 0.610). Los niveles de actividad paraoxonasa no fueron significativamente diferentes entre los grupos control y con infarto (450 vs. 348 Ul/mL, *p*= 0.093). Mientras que los niveles de actividad arilesterasa fueron significativamente diferentes entre los grupos estudiados (90 vs. 70 Ul/mL, *p*= 0.001), sin embargo, al ajustarla por regresión lineal múltiple, dejo de ser significativa.

**Conclusión:**

Los polimorfismos Q192R y L55M, y la actividad paraoxonasa de la PON1 no son factores de riesgo para el infarto cerebral aterotrombótico en este estudio.

## Introduction

The ischemic cerebrovascular disease (CVD) or cerebral stroke, is caused by the interruption of cerebral blood flow caused by a thrombus promoted by atherosclerosis or cardioembolism ^1^. Atherothrombotic cerebral infarction is the most common subtype of cerebral infarctions, having an approximate frequency of 37% ^2^ being the clinical consequence of the atheromatous disease. Multiple factors induce atherogenesis, mainly oxidized- low density lipoproteins (ox-LDL), which trigger an immune response in the wall of the vessel participating in the development of atherosclerosis ^3^. LDL oxidation degree depends on the balance of oxidants/antioxidants agents and the existing LDL concentration ^4^. On the other hand, it is known that high density lipoproteins (HDL) have an anti-atherosclerotic role, since they metabolize ox-LDL in the arterial wall, transporting them to the liver for their elimination ^5^ ^,^ ^6^. This capacity of the HDL is due to the association of enzymes such as paraoxonase-1 (PON1) and acetylhydrolase of the platelet activating factor (PAF-AH) which decrease LDL peroxidation ^4^ ^,^ ^7^. 

PON1 is a 355 amino acids enzyme, synthesized by the liver and secreted into the bloodstream, where it is associated with HDL ^8^ ^,^ ^9^. PON1 presents a polymorphism at position 192 of the coding region, that is characterized by a substitution of a glutamine (Q) by an arginine (R) and another one at position 55, where there is a change from a Leucine (L) to a methionine (M) ^10^. Q192R polymorphism affects the ability to hydrolyze organophosphorus compounds such as paraoxon ^11^. The two Q192R polymorphism allozymes have different affinities and catalytic activities towards several substrates ^12^. These polymorphisms also modify PON1 ability to protect LDLs from oxidation.

From the observed effect of common polymorphisms of PON1 on the protection by HDL against the oxidative modification of LDL, it has been suggested that homozygotes for Q and for M of PON1 could be less susceptible of developing atherosclerotic diseases, and homozygotes for R and L could be more prone to develop those diseases ^13^. The frequent association of RR genotype of PON1 192 with the risk of cardiovascular diseases reflects a diminished efficiency in the metabolism of oxidized lipids and/or less stability of this alloenzyme compared with QQ genotype ^14^. In this context, PON1 Q192R and PON1 L55M polymorphisms, as well as enzymatic activity of PON1, have been considered a tool that can contribute to the risk estimation of atheromatous diseases. Thus, the aim of this study was to investigate the relationship between PON 1 genetic polymorphisms and ischemic CVD of atherosclerotic etiology. 

## Materials and Methods

 A study of cases and controls was conducted by the National Institute of Neurology and Neurosurgery (INNN) in Mexico City, during the year 2005. The participants or relatives signed an informed consent and answered a questionnaire (demographic, clinical and life styles data) and provided a venous blood sample for biochemical and molecular analysis. The case group were hospitalized subjects between 35 and 85 years of age, with a recent diagnostic of ischemic CVD atherothrombotic type in acute phase. The control group was subjects between 35 and 75 years of age who passed the established tests for blood donation in the same hospital. The study was approved by the INNN Bioethics Committee (No. 67/01).

For biochemical assays the Hitachi 912 autoanalyzer (Roche, Basel, Switzerland) was used. The total serum cholesterol (TC) and triglycerides (TG) determination were carried out with CHOD-PAP, HDL-C with HDL-C plus third generation and LDL-C with LDL-C second generation (Roche Diagnostic).

Paraoxonase activity was determined in serum by modifying the Eckerson method ^15^. The enzyme-substrate reaction was initiated by the addition of 20 µL of serum plus 980 µL of buffer pH 8 (Tris 10 mM, 1 mM CaCl2, 2.6 M NaCl µL, 1 mM of paraoxon). The rate of hydrolysis was determined with a UV-VIS spectrophotometer (Varian Cary 50, Varian Inc., Palo Alto, CA) by measuring the hydrolysis product (p-nitrophenol) at a wavelength of 412 nm. Increases in A_412_ continued for 5 min (a molar extinction coefficient of 17,100 M^-1^ cm^-1^ was used) ^16^. For the arilesterase activity, 2,995 µL of phenyl acetate (1 mM) was used as a substrate in a pH 8 buffer (Tris 10 mM CaCl_2_ 1 mM) and 5 µL of serum. The hydrolysis rate was determined by measuring the product (phenol) at 270 nm wavelength, monitoring it for 3 min and registering the value before and after the incubation was obtained. Absorbance was adjusted based on the molar extinction coefficient 1,310 M cm^−1 (^ ^15^.

To determine PON1-Q192R and PON1-L55M genetic polymorphisms, genomic DNA was extracted from whole blood using a commercial Kit (AquapureTM Genomic DNA kit, Bio-rad Laboratories, Hercules, CA). The identification of PON1 genotypes was carried out by PCR-RFLP ^17^, with further digestion of the amplified products with *BspPI* restriction enzymes (Fermentas) for PON1-Q192R and *Hin1II* (Fermentas) for PON1-L55M. The digested fragments were separated by electrophoresis and visualized in polyacrylamide gels at 7.5% and 20% for PON1-L55M (two fragments of 126 and 44 pb) and PON1-Q192R (two fragments of 66 and 33 pb), respectively. The laboratory personnel carried out the samples genotyping in a blind test which included control samples (identified previously by sequencing) and the experimental samples validation. The concordance with the control samples was 100%.

The categorical variables were compared by using Chi square test. The variables with normal distribution were compared by a Students-t or ANOVA. Those non normal variables were analyzed with the U test of Mann-Whitney and were further transformed to logarithmic values to carry out the regression tests. The variables were evaluated in a simple linear regression, those with a *p*-value of less than or equal to 0.1 were included in a saturated model of multiple linear regression, eliminating those that lost statistical significance. Age and sex were maintained for being potentially confounding. The data were analyzed by using the SPSS^®^ statistical program version 13, with a statistical significance of 0.05.

## Results

****The present study included 28 cases of atherothrombotic cerebral infarction and 29 controls. Table 1 show that age, sex and alcohol and tobacco consumption were different between cases and controls. The clinical parameters of BMI, serum lipids (with the exception of triglycerides) and hypercholesterolemia were similar between both groups.

The genotypes frequencies of Q192R and L55M polymorphisms of PON1 (Table 1) were not statistically different (*p*= 0.786 and *p*= 0.282, respectively) between the control and case groups. Subsequently, the allelic distributions of Q/R and L/M for the Q192R and L55M polymorphisms respectively, were compared and no differences were found. 

**Table 1:** Clinical and anthropometric characteristics and allele/genotype frequencies of the study groups.

	Controls (n= 28)	Cases (n= 29)	p*
Age (years) †	48 (43-54.5)	61 (53-71.5)	0.001
Male/female	21/8	13/17	0.024
BMI (kg/m^2^) †	27. 12±0. 80	27. 77±0. 78	0.568
TG (mg/dL) †	31.02	23.41	0.073
Total-C (mg/dL) †	213. 62±10. 06	191. 98±9. 05	0.116
HDL-C (mg/dL) †	28.18	22.82	0.193
LDL-C (mg/dL) †	124. 41±9. 97	124. 22±6. 31	0.987
HTA, n (%)	2 (6.9)	16 (55.2)	< 0.001
DM, n (%)	0 (0.0)	9 (31.0)	0.001
Tobacco consumption, n (%)	2 (6.9)	8 (27.6)	0.037
Alcohol consumption, n (%)	0 (0.0)	8 (28.6)	0.002
Genotype PON1-L55M	Frequency (%)	Frequency (%)	
LL	66.7	69	0.282
LM	29.6	17.2	
MM	3.7	13.8	
Allele L	81.5	77.6	0.610
Allele M	18.5	22.4	
Genotype PON1-Q192R			
QQ	21.4	27.6	0.786
QR	50.0	41.4	
RR	28.6	31.0	
Allele Q	46.4	48.3	0.843
Allele R	53.6	51.7	

†Continuous variables are expressed as the mean ± standard error or median (25 quartile- 75 quartile).

* Value significance for Students t or Chi square test.

Triglycerides (TG), HDL cholesterol (HDL-C) and LDL cholesterol (LDL-C), the value of significance was for U-Mann Whitney. Total-C: total cholesterol.

**Figure 1:**
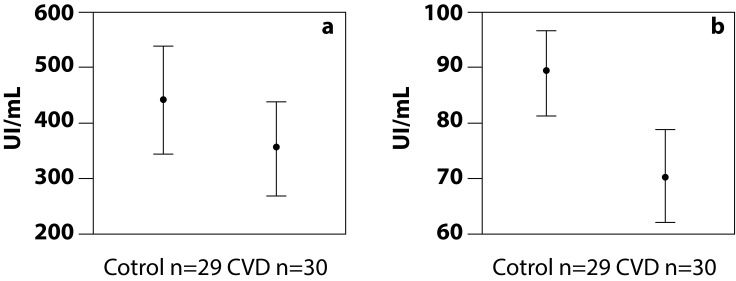
Paraoxonase and arylesterase activities of serum PON1. Bars represent the confidence interval at 95%; (a) paraoxonase activity, *p*= 0.093 and (b) arylesterase activity, *p*= 0.001, by student t test.

The paraoxonase activity of PON1 was greater in the control group (450.27 UI/mL) than in the case group (348.35 UI/mL), although the difference was not statistically significant (*p*= 0.093). This was similar to the arylesterase activity of PON1, which was greater in the control group (89.96 IU/mL) than in the case group (69.63 IU/mL), being this difference statistically significant (*p*= 0.001) (Fig. 1). 

In the simple regression analysis for paraoxonase activity (Table 2), it was observed that such activity was explained by serum triglyceride, by arylesterase, by both polymorphisms and by the (cases *vs* controls). In the multiple regression model, it was demonstrated that age, arylesterase and Q192R polymorphism, were the factors that determined in a significant and independent way paraoxonase activity variation in 78.5%. It was also found that diagnosis (*p* = 0.85 in the saturated model, data not shown), did not influence paraoxonase activity of the subjects. 

**Table 2:** Linear regression analysis for paraoxonase activity.

Simple regression	β1	Adjusted r^**2**^	*p*
Age	-450.54	0.022	0.136
Sex	-38.85	-0.011	0.532
BMI	9.41	0.008	0.232
TG *	201.54	0.035	**0.096**
Total-C	0.77	0.007	0.252
HDL-C	105.59	-0.018	0.731
LDL-C	-0.026	-0.022	0.975
Hypercholesterolemia	37.98	-0.013	0.594
HTA	-37.70	-0.012	0.576
DM	-51.78	-0.011	0.548
Tobacco consumption	39.78	-0.014	0.630
Alcohol consumption	28.01	-0.017	0.756
Statins	-36.63	-0.017	0.769
Diagnosis *	-101.92	0.032	**0.093**
Arylesterase activity *	2.13	0.030	**0.100**
PON192 *	232.72	0.563	**< 0.0001**
PON55*	-127.73	0.127	**0.004**
Multiple regression ^†^	β1	Adjusted r^2^	*p*
Age	-327.80		0.0370
Arylesterase activity	3.993	0.785	< 0.0001
PON192	271.43		< 0.0001

*Variables with a *p* value ≤ 0.1.

† statistically significant variables (*p* < 0.05).

TG: triglycerides, Total-C: total cholesterol, HDL-C: HDL- cholesterol, LDL-C: LDL- cholesterol, β1: slope value and r^2^ adjusted: coefficient of correlation.

For arylesterase activity (Table 3), in the simple regression analysis, the variables of diagnosis, age, total cholesterol, alcohol consumption and Q192R polymorphism were related with the activity. In the multiple regression analysis, age, alcohol consumption and total cholesterol (the latter with a marginal significance) determined in a significant and independent way the arylesterase activity variation, although they only explained it in a 27.6%. The statistical difference obtained from the arylesterase activity between control and case groups was not longer significant upon adjustment during the multiple linear regression.

**Table 3:** Linear regression analysis for arylesterase activity.

Simple regression	β1	Adjusted r^**2**^	*p*
Age *	-89.70	0.134	0.003
Sex	-2.68	-0.014	0.669
BMI	-0.028	-0.019	0.970
TG	13.27	0.010	0.225
Total-C*	0.120	0.062	0.042
HDL-C	21.01	-0.008	0.433
LDL-C	0.092	0.012	0.214
Hypercholesterolemia	1.43	-0.017	0.841
HTA	-6.28	-0.002	0.353
DM	-5.12	-0.011	0.554
Tobacco consumption	-9.66	0.007	0.242
Alcohol consumption *	-26.41	0.173	0.001
Statins	-11.45	-0.002	0.354
Diagnosis *	-20.33	0.175	0.001
PON192 *	-9.08	0.064	0.032
PON55	2.57	-0.013	0.601
Multiple regression ^†^	β1	r^2^ adjusted	*p*
Age	-64.74		0.019
Total-C	0.103	0.276	0.051
Alcohol consumption	-15.578		0. 041

* Variable with a *p* value ≤0. 1.

† Statistically significant variables (*p* < 0.05).

TG: triglycerides, Total-C: total cholesterol, HDL-C: HDL cholesterol, LDL-C: LDL cholesterol, β1: value of the slope and r^2^ adjusted: correlation coefficient.

## Discussion

In this research, the analysis of the allelic distribution and the corresponding genotypes for Q192R and L55M of the PON1 polymorphisms showed an absence of risk due to these genetic factors for atherothrombotic CVD, similar to the ones found in other populations, even with a larger sample, for risk of coronary heart disease ^18^ and cerebral infarction ^19^. However, there are still conflicting results, since in a meta-analysis, it was found that there is a small increase in suffering cerebral infarction in people presenting the R allele of Q192R polymorphism ^20^. An optimal analysis of the relationship between PON1 polymorphisms and CVD in our population could be carried out, increasing the number of individuals in the control and case groups,

Regarding PON1 activity, this study demostrated that control subjects and the subjects with infarction had no differences in the paraoxonase and arylesterase activities, thus, the level of activity of this enzyme did not represent a risk for the atherothrombotic infarction. The linear regression analysis allowed us to know that age is one of the factors which have influence in the levels of the paraoxonase and arilesterase activities of PON1. While alcohol consumption and cholesterol levels were factors which also had an independent effect regardless of age on the arilesterase activity, suggests to consider such variables in future studies in order to confirm its effect on PON activity.

The arylesterase activity has been used as a measure of PON1 level ^8^ ^,^ ^14^ ^,^ ^21^. Therefore, the homogeneous distribution that was found in the arilesterase activities among QQ, QR and RR genotypes suggests similar levels of this enzyme. Differences found in paraoxonase activity were not dependent on PON1 level but on Q192R genotype, as it was proposed when the polymorphism was discovered for the first time ^15^.

Comparing our control group with the reference group in a study carried out in the United Kingdom ^18^, we found a notable difference between paraoxonase activities (450.27 vs. 214.6 Ul/mL) where Mexican subjects presented the higher activity. After grouping by genotype the same comparison, the paraoxonase activities in the control subjects QQ, QR, and RR (122.3, 463.9 and 695.1 UI/mL, respectively) were also higher than in the respective genotype (116, 226, and 396.4 UI/mL) of the control group in the United Kingdom ^18^ (the taken data were the reported medians). It is possible that such differences are explained by a different exposure among both populations such as environmental factors and other factors which regulate the PON1 expression, stimulate or inhibit its activity. A comparison must be done between both control groups.

One of the limitations of this work was the samples size, as well as the fact that it was not possible to assess the effect of other variables such as food habits, drug therapies or comorbid diseases that could influence arilesterase and paraoxonase activities of PON-1.

## Conclusion

In this study where Mexican subjects were included, Q192R and L55M polymorphisms, paraoxonase and arilesterase activities from PON1 are not risk factors for atherothrombotic cerebral infarction.
