# Supplementary material for: Paraoxonase-1 polymorphisms and cerebral ischemic stroke: a pilot study in mexican patients
Source: Colomb Med (Cali). 2018 Sep 30;49(3):223–7. doi: 10.25100/cm.v49i2.2217 (PMC6220488; doi:10.25100/cm.v49i2.2217)
Supplement: Related texts

# Research article: Caracterización polimórfica de la paraoxonasa-1 en el infarto cerebral aterotrombótico de una poblacion mexicana: estudio piloto

## Introducción

La enfermedad vascular cerebral (EVC) isquémica o infarto cerebral, es causada por la interrupción del flujo sanguíneo cerebral ocasionada por un trombo promovido por la ateroesclerosis o la cardioembolia ^1^. El infarto cerebral aterotrombótico es el subtipo más común de los infartos cerebrales, tiene una frecuencia aproximada del 37% ^2^ y es la consecuencia clínica de la enfermedad ateromatosa. Múltiples factores inducen la aterogénesis, principalmente lipoproteínas de baja densidad (LDL) oxidadas, las cuales desencadenan una respuesta inmunológica en la pared del vaso sanguíneo y participan en el desarrollo de la ateroesclerosis ^3^. El grado de oxidación de las LDL depende del balance de agentes oxidantes/antioxidantes y de la cantidad de LDL existente ^4^. Por otro lado, se sabe que las lipoproteínas de alta densidad (HDL) tienen un papel anti-ateroesclerótico, ya que metabolizan moléculas de LDL oxidadas de la pared arterial, transportándolas al hígado para su eliminación ^5^^,^^6^. Esta capacidad de la HDL es debida a la asociación de enzimas como la paraoxonasa 1 (PON1) y la acetilhidrolasa del factor activador de plaquetas (PAF-AH) que disminuyen la peroxidación de las LDL ^4^^,^^7^.

PON1 es una enzima de 355 aminoácidos, sintetizada en el hígado y secretada en la sangre, donde se encuentra unida a las HDL ^8^^,^^9^. PON1 presenta, un polimorfismo en la posición 192 en la región codificante, que se caracteriza por una sustitución de una glutamina (Q) por una arginina (R) y otra en la posición 55, donde se evidencia un cambio de una leucina (L) por una metionina (M) ^10^. El polimorfismo Q192R afecta la capacidad para hidrolizar los compuestos organofosforados como el paroxón ^11^. Las dos aloenzimas del polimorfismo Q192R tienen afinidades y actividades catalíticas diferentes frente a varios sustratos ^12^. Estos polimorfismos también modifican la capacidad de la PON1 para proteger *in vitro* a las LDLs de la oxidación.

A partir del efecto observado de los polimorfismos comunes de la PON1 sobre la protección por las HDL ante la modificación oxidativa de las LDL, se ha sugerido que los individuos homocigotos para Q y para M de la PON1 podrían ser menos susceptibles a desarrollar enfermedades de origen ateroesclerótico, y que individuos homocigotos para R y para L podrían ser más propensos a desarrollar dichas enfermedades ^13^. La frecuente asociación del genotipo RR de PON1-192 con el riesgo de enfermedades cardiovasculares refleja una eficiencia disminuida en el metabolismo de lípidos oxidados y/o una menor estabilidad de esta aloenzima, comparada con el genotipo QQ ^14^. En este contexto, los polimorfismos Q192R y L55M, así como la actividad enzimática de la PON1, se han considerado una herramienta que puede contribuir a la estimación del riesgo de padecer las enfermedades referidas. Por lo tanto, el objetivo del presente estudio fue investigar la relación de los polimorfismos genéticos de PON1 con la EVC isquémica, de etiología ateroesclerosa.

## Materiales y Métodos

Se realizó un estudio de casos y controles en el Instituto Nacional de Neurología y Neurocirugía (INNN), de la Ciudad de México, durante el año 2005. Los participantes o familiares responsables, firmaron un consentimiento informado, contestaron un cuestionario (datos demográficos, clínicos y de estilos de vida) y proporcionaron una muestra de sangre venosa para análisis bioquímicos y moleculares. Los casos fueron sujetos hospitalizados de 35 a 85 años de edad con diagnóstico reciente de EVC isquémica de tipo aterotrombótica en fase aguda. Los controles fueron sujetos entre 35 y 75 años que pasaron las pruebas establecidas para la donación de sangre del mismo hospital. El estudio fue aprobado por el Comité de Bioética del INNN (No. 67/01).

Para los ensayos bioquímicos se empleó el autoanalizador Hitachi 912 (Roche, Basel, Switzerland). La determinación de colesterol total sérico (TC) y triglicéridos TG se realizó con CHOD-PAP, HDL-C con HDL-C plus 3ª generación y LDL-C con LDL-C 2ª generación, todas de Roche Diagnostic.

La actividad paraoxonasa se determinó en suero a través de modificaciones al método de Eckerson ^15^, 20 µL de suero más 980 µL de amortiguador pH 8 (Tris 10 mM, CaCl2 1 mM, 2.6 M de NaCl µL, 1 mM de paraoxón), midiendo el producto de hidrólisis (p-nitrofenol) a una longitud de onda de 412 nm durante 5 min, (se utilizó un coeficiente de extinción molar de 17,100 ^16^. Para la actividad arilesterasa se utilizaron 2,995 µL de acetato de fenilo 1 mM en un amortiguador pH 8 (Tris 10 mM, CaCl2 1 mM) y 5 µL de suero. La velocidad de hidrólisis se determinó midiendo el producto (fenol) a una longitud de onda de 270 nm, se monitoreó durante 3 min y se tomó el valor antes y después de la incubación. Se ajustó la absorbancia en base a su coeficiente de extinción molar de 1,310 M^−1^cm^−1 (^^15^.

Para la determinación de los polimorfismos genéticos de PON1-Q192R y PON1-L55M, el DNA genómico se extrajo a partir de sangre completa mediante un Kit comercial (AquapureTM Genomic DNA kit, Bio-rad Laboratories, Hercules, CA). La identificación de los genotipos de PON1 se realizó por PCR ^17^, con posterior digestión de los productos amplificados con enzimas de restricción *BspPI* (Fermentas) para PON1-Q192R y *Hin1II* (Fermentas) para PON1-L55M. Los fragmentos digeridos fueron separados por electroforesis y visualizados en geles de poliacrilamida al 7.5% y 20% para PON1-L55M (dos fragmentos de 126 y 44 pb) y PON1-Q192R (dos fragmentos de 66 y 33 pb), respectivamente. El personal de laboratorio llevó a cabo la genotipificación de las muestras en una prueba ciega que incluyó muestras control (identificadas previamente mediante secuenciación) y las muestras experimentales para su validación. La concordancia con las muestras control fue del 100%.

Las variables categóricas se compararon mediante Chi cuadrado. Las variables con distribución normal se compararon mediante t de Student o ANOVA, aquellas variables no normales se analizaron con la prueba U de Mann-Whitney y posteriormente se transformaron a valores logaritmos para realizar las pruebas de regresión. Se evaluaron las variables en una regresión lineal simple, aquellas con un valor de *p* menor o igual a 0.1, se incluyeron en un modelo saturado de regresión lineal múltiple, eliminando aquellas que perdían significancia estadística, se mantuvieron edad y sexo por ser potenciales confusoras. Los datos fueron analizados con el programa estadístico SPSS® versión 13, con una significancia estadística de 0.05.

## Resultados

En el presente trabajo se incluyeron 28 casos de infarto cerebral aterotrombótico y 29 controles. En la Tabla 1 se observa que la edad, el sexo y el consumo de alcohol y tabaco fueron distintos entre casos y controles. Los parámetros clínicos de IMC, lípidos séricos (a excepción de triglicéridos) e hipercolesterolemia fueron similares entre ambos grupos.

**Tabla 1:** Características clínicas, antropométricas y frecuencias alélicas y genotípicas de los grupos de estudio.

	Controles (n= 28)	Casos (n= 29)	p*
Edad (años) †	48 (43-54.5)	61 (53-71.5)	0.001
Hombre/mujer	21/8	13/17	0.024
IMC (kg/m2) †	27.12±0.80	27.77±0.78	0.568
TG (mg/dL) †	31.02	23.41	0.073
C-Total (mg/dL) †	213.62±10.06	191.98±9.05	0.116
C-HDL (mg/dL) †	28.18	22.82	0.193
C-LDL (mg/dL) †	124.41±9.97	124.22±6.31	0.987
HTA, n (%)	2(6.9)	16(55.2)	< 0.001
DM, n (%)	0(0.0)	9(31.0)	0.001
Consumo de tabaco n (%)	2(6.9)	8(27.6)	0.037
Consumo de alcohol, n (%)	0(0.0)	8(28.6)	0.002
**Genotipo PON1-L55M**	**Frequency (%)**	**Frequency (%)**	
LL	66.7	69	0.282
LM	29.6	17.2	
MM	3.7	13.8	
Alelo L	81.5	77.6	0.610
Alelo M	18.5	22.4	
Genotipo PON1-Q192R			
QQ	21.4	27.6	0.786
QR	50.0	41.4	
RR	28.6	31.0	
Alelo Q	46.4	48.3	0.843
Alelo R	53.6	51.7	

†Las variables continuas están expresadas como la media ±error estándar o mediana (cuartil 25-cuartil 75).

* Valor de la significancia para t de Student o Chi cuadrado.

Para triglicéridos (TG), colesterol de HDL (C-HDL) y colesterol de LDL (C-LDL) el valor de la significancia fue para U de Mann Whitney. C-Total: colesterol total.

Las frecuencias de las tres clases genotípicas correspondientes a los polimorfismos Q192R y L55M de la PON1 (Tabla 1) no fueron estadísticamente diferentes (*p*= 0.786 y *p*= 0.282, respectivamente) entre el grupo control y de casos, posteriormente se compararon las distribuciones alélicas de Q/R para el polimorfismo Q192R y L/M para el polimorfismo L55M las cuales tampoco fueron diferentes entre ambos grupos.

La actividad paraoxonasa de la PON1 fue mayor en el grupo control (450.27 UI/mL) en relación al grupo de casos (348.35 UI/mL), aunque no fue estadísticamente significativo (*p*= 0.093). Situación similar a la actividad arilesterasa de la PON1, que fue mayor en el grupo control (89.96 UI/mL) en relación al grupo de casos (69.63 UI/mL), siendo esta diferencia estadísticamente significativa (*p*= 0.001) (Fig. 1).

**Figura 1:** Actividades paraoxonasa y arilesterasa de la PON1 en suero**.** Las barras representan el intervalo de confianza al 95%. *p*= 0.093 para paraoxonasa (**a**) y *p*= 0.001 para arilesterasa (**b**), por t de Student.

En el análisis de regresión simple para la actividad paraoxonasa (Tabla 2), se observa que dicha actividad fue explicada por los niveles séricos de triglicéridos, por la actividad arilesterasa, por ambos polimorfismos y por el diagnóstico (casos *vs* controles). En el modelo de regresión múltiple se demostró que la edad, la actividad arilesterasa y el polimorfismo Q192R, fueron los factores que determinaron de manera significativa e independiente en un 78.5% la variación de la actividad paraoxonasa. También se encontró que el diagnóstico (p=0.85 en el modelo saturado, dato no mostrado), no influyó en la actividad paraoxonasa de los sujetos de este estudio.

**Tabla 2:** Análisis de regresión lineal para la actividad paraoxonasa.

Regresión simple	β1	r2 ajustado	*p*
Edad	-450.54	0.022	0.136
Sexo	-38.85	-0.011	0.532
IMC	9.41	0.008	0.232
TG*	201.54	0.035	**0.096**
C-Total	0.77	0.007	0.252
C-HDL	105.59	-0.018	0.731
C-LDL	-0.026	-0.022	0.975
Hipercolesterolemia	37.98	-0.013	0.594
HTA	-37.70	-0.012	0.576
DM	-51.78	-0.011	0.548
Tabaquismo	39.78	-0.014	0.630
Alcoholismo	28.01	-0.017	0.756
Estatinas	-36.63	-0.017	0.769
Diagnóstico*	-101.92	0.032	**0.093**
Actividad arilesterasa*	2.13	0.030	**0.100**
PON192*	232.72	0.563	**< 0.0001**
PON55*	-127.73	0.127	**0.004**
**Regresión múltiple** ^†^	**β1**	**r2 ajustado**	*p*
Edad	-327.80		0.0370
Actividad arilesterasa	3.993	0.785	< 0.0001
PON192	271.43		< 0.0001

*Variables con un valor p≤ 0.1.

†variables estadísticamente significativas (*p* <0.05).

TG: triglicéridos, C-Total: colesterol total, C-HDL: colesterol de HDL, C-LDL: colesterol de LDL, β1: valor de la pendiente y r^2^ ajustado: coeficiente de correlación.

En relación a la actividad arilesterasa (Tabla 3), en el análisis de regresión simple, las variables de diagnóstico, edad, colesterol total, alcoholismo y el polimorfismo Q192R estuvieron relacionados con la actividad. En el análisis de regresión múltiple, la edad, el consumo de alcohol y el colesterol total (este último con una significancia marginal) determinaron de manera significativa e independiente la variación de la actividad arilesterasa, aunque sólo lo explicaron en un 27.6%. La d iferencia estadística obtenida de la actividad arilesterasa entre controles y casos dejo de ser significativa al ajustarla durante la regresión lineal múltiple.

**Tabla 3:** Análisis de regresión lineal para la actividad arilesterasa.

Regresión simple	β1	r2 ajustado	*p*
Edad*	-89.70	0.134	0.003
Sexo	-2.68	-0.014	0.669
IMC	-0.028	-0.019	0.970
TG	13.27	0.010	0.225
C-Total*	0.120	0.062	0.042
C-HDL	21.01	-0.008	0.433
C-LDL	0.092	0.012	0.214
Hipercolesterolemia	1.43	-0.017	0.841
HTA	-6.28	-0.002	0.353
DM	-5.12	-0.011	0.554
Tabaquismo	-9.66	0.007	0.242
Alcoholismo*	-26.41	0.173	0.001
Estatinas	-11.45	-0.002	0.354
Diagnóstico*	-20.33	0.175	0.001
PON192*	-9.08	0.064	0.032
PON55	2.57	-0.013	0.601
**Regresión múltiple** ^†^	**β1**	**r2 ajustado**	**p**
Edad	-64.74		0.019
C-Total	0.103	0.276	0.051
Alcoholismo	-15.578		0.041

*Variables con un valor *p* ≤0.1.

†variables estadísticamente significativas (*p* <0.05).

TG: triglicéridos, C - Total: colesterol total, C-HDL: colesterol de HDL, C-LDL: colesterol de LDL, β1: valor de la pendiente y r^2^ ajustado: coeficiente de correlación.

## Discusión

En la presente investigación, el análisis de la distribución alélica y de los genotipos correspondientes a los polimorfismos Q192R y L55M de la PON1 demostró una ausencia de riesgo por estos factores genéticos para la EVC aterotrombótica, similar a lo encontrado en otras poblaciones, aún con una muestra mayor, para el riesgo de enfermedad coronaria ^18^ e infarto cerebral ^19^. Sin embargo, existen aún resultados contradictorios, ya que en un meta- análisis realizado se encontró que hay un pequeño incremento a padecer infarto cerebral en personas que presentan el alelo R del polimorfismo Q192R ^20^. Posiblemente, aumentando el número de individuos en los grupos control y de casos se realizaría un óptimo análisis de la relación entre los polimorfismos de la PON1 y la EVC en nuestra población.

Respecto a la actividad de la PON1, en este estudio se demostró que los sujetos control y los sujetos con infarto no tuvieron diferencias en las actividades paraoxonasa y arilesterasa, por lo que el nivel de actividad de esta enzima no representó un riesgo para el infarto aterotrombótico. El análisis de regresión lineal permitió conocer que la edad es uno de los factores que influyen en los niveles de las actividades paraoxonasa y arilesterasa de la PON1. Mientras que el consumo de alcohol y los niveles de colesterol fueron factores que también tuvieron un efecto independiente de la edad sobre la actividad arilesterasa, lo cual sugiere considerar dichas variables en futuros estudios para confirmar su efecto sobre la actividad de PON, así como elucidar el mecanismo por el cual la disminuye.

La actividad arilesterasa se ha utilizado como una medida del nivel de PON1 ^8^^,^^14^^,^^21^, por lo tanto, la distribución homogénea que se encontró de las actividades arilesterasa entre los genotipos QQ, QR y RR sugiere una presencia similar de esta enzima y que las diferencias encontradas en la actividad paraoxonasa entre los subgrupos genotípicos no fueron dependientes de la cantidad de la PON1 sino del genotipo, como fue propuesto cuando se descubrió este polimorfismo por primera vez ^15^.

Comparando nuestro grupo control con el grupo de referencia de un estudio realizado en el Reino Unido ^18^, encontramos una diferencia notable entre las actividades paraoxonasa (450.27 vs. 214.6 Ul/mL), presentando los sujetos mexicanos el promedio más alto de esta actividad. En este mismo cotejo, después de agrupar por genotipo se observó lo mismo, esto es, las actividades paraoxonasa en los sujetos control QQ, QR y RR (122.3, 463.9 y 695.1 UI/mL, respectivamente) fueron en promedio más altas que en los respectivos genotipos (116, 226 y 396.4 UI/mL) del grupo control del estudio en el Reino Unido ^18^ (los datos tomados fueron las medianas reportadas). Tal vez estas diferencias son explicadas por una exposición diferente entre ambas poblaciones a factores ambientales conocidos y a otros que aun no se conocen, los cuales regulan la expresión de la PON1 o estimulan o inhiben su actividad, habría que hacer una comparación entre ambos grupos control.

Una de las limitaciones de este trabajo fue el tamaño de muestra, así como también el hecho de que no fue posible evaluar el efecto de otras variables como hábitos alimentación, terapias medicamentosas o enfermedades concomitantes que podrían influir en las actividades arilesterasa y paraoxonasa de la PON-1.

## Conclusión

Los polimorfismos Q192R y L55M, las actividades paraoxonasa y arilesterasa de PON1 no son factores de riesgo para el infarto cerebral aterotrombótico en este estudio donde se incluyeron sujetos mexicanos.
